# Future climate and demographic changes will almost double the risk of schistosomiasis transmission in the Lake Victoria Basin

**DOI:** 10.1016/j.onehlt.2025.101148

**Published:** 2025-07-18

**Authors:** Zadoki Tabo, Rapheal Wangalwa, Marcellin Rwibutso, Lutz Breuer, Christian Albrecht

**Affiliations:** a Centre for International Development and Environmental Research (ZEU), Justus Liebig University Giessen, Senckenbergstrasse 3, 35390 Giessen, Germany; b Institute for Landscape Ecology and Resources Management (ILR), Research Centre for Biosystems, Land Use and Nutrition (iFZ), Justus Liebig University Giessen, Heinrich-Buff-Ring 26, 35392 Giessen, Germany; c Department of Animal Ecology and Systematics, Justus Liebig University Giessen, Heinrich-Buff-Ring 26 (iFZ), 35392 Giessen, Germany; d Department of Biology, Mbarara University of Science and Technology (MUST), P. O. Box 1410, Mbarara, Uganda

**Keywords:** One Health, *Biomphalaria*, *Bulinus*, Vector snails, Ecological ensemble modeling, *Schistosoma* transmission risks

## Abstract

**Background:**

The Lake Victoria Basin (LVB), supporting millions of people across Uganda, Kenya, Tanzania, Rwanda, and Burundi, is a critical freshwater ecosystem. However, it faces significant One Health challenges, notably urogenital and intestinal schistosomiasis, diseases transmitted by *Bulinus* and *Biomphalaria* snails, respectively. Climate, topography, environmental and demographic drivers influence snail habitat suitability and distribution, potentially increasing schistosomiasis risks for communities dependent on agriculture, fishing, and water-related livelihoods.

**Methods:**

This study applied ecological ensemble modeling (Random Forest, XGBoost, and MaxEnt) to identify key drivers of habitat suitability, assessing current and future climate scenarios under Shared Socioeconomic Pathways, and overlaying habitat suitability and population density to quantify human exposure risks associated with *Biomphalaria* and *Bulinus* snails. Snail occurrence data were sourced from biodiversity databases, field surveys, and literature. Predictor variables included climatic (temperature, precipitation), topographic (elevation, slope, proximity to water bodies), and environmental (vegetation index, soil composition) features.

**Findings:**

For *Bulinus*, habitat suitability increased with higher precipitation and elevation but decreased with rising vegetation index NDVI, silt content, and temperature seasonality as key drivers. Similarly, *Biomphalaria* suitability improved with higher precipitation and elevation but declined with increasing NDVI, slope, and temperature seasonality. Currently, *Biomphalaria* and *Bulinus* are primarily concentrated around Lake Victoria, with 17 % and 14 % of the area suitable for their habitat, a figure projected to increase to 21 % and 18 % by 2050. By 2050, medium -risk zones for intestinal schistosomiasis are expected to almost double from 13 % to 22 %, while those for urogenital schistosomiasis are projected to more than double from 8 % to 18 % of the total area. This study predicts a rising schistosomiasis risk across the LVB, emphasizing the need for targeted interventions. A proactive One Health approach, integrating environmental management, strategic disease control, and policy adaptation, is vital to reducing future risks and protecting vulnerable communities.

## Highlights

• Ecological modeling reveals environmental, topographical and climatic drivers forschistosomiasis.
• Climate changes influence the spatial distribution of *Biomphalaria* and *Bulinus* snails, influencing disease risk.
• Geospatial analysis integrates human population data to assess future schistosomiasis exposure risks.
• Climate and demographic projections may nearly double schistosomiasis high-risk zones in the Lake Victoria Basin.
• A proactive One Health approach is key to managing schistosomiasis via environment and policy actions.

## Graphical abstract

**Figure:**
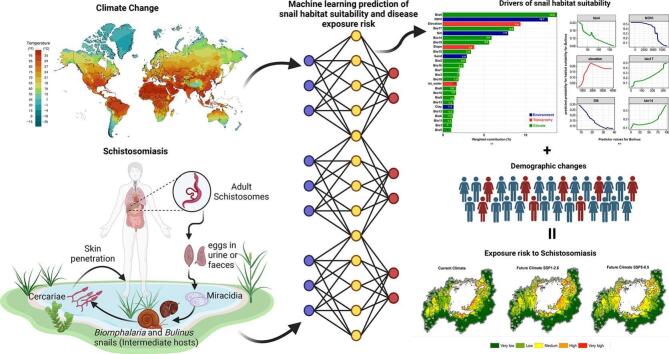

## 1 Introduction

Schistosomiasis, or bilharzia, is a significant yet neglected tropical disease caused by *Schistosoma* blood flukes, affecting both humans and animals [1]. It remains one of the most widespread waterborne diseases, with over 240 million infections and 1.53 million disability-adjusted life years (DALYs) lost annually [2,3]. Sub-Saharan Africa bears 90 % of the global burden, with an estimated 280,000 deaths per year [2,4]. Socio-economic impacts include reduced productivity, hindered childhood development, and increased healthcare costs, thereby straining public health systems and economic stability.

Transmission occurs in freshwater habitats where specific snails serve as intermediate hosts (IH), releasing infectious cercariae that penetrate human skin upon water contact. Inside the body, larvae mature, pair, and produce eggs, some of which are excreted via urine or feces into water sources. Under suitable conditions, eggs hatch into miracidia, infecting snails and completing the cycle [1]. The primary species responsible for schistosomiasis disease burden in Africa are *Schistosoma mansoni*, which causes intestinal schistosomiasis and is transmitted by basically all species of the genus *Biomphalaria*, and *Schistosoma haematobium*, which causes urogenital schistosomiasis and is transmitted by selected snails of the genus *Bulinus* [1,5]. Transmission occurs where ecological conditions support snail vectors, with high-risk activities such as bathing, domestic water use, fishing, and agriculture increasing exposure, especially in areas with poor sanitation [2,6].

Currently, no vaccine exists for schistosomiasis, though research is ongoing [7]. Praziquantel drug remains the primary treatment [3], but challenges like reinfection [8] and potential drug resistance threaten control efforts [9]. Recognizing these limitations, One Health approaches are gaining traction alongside medical interventions [9]. The WHO 2030 roadmap highlights One Health strategies, emphasizing snail control, and has become a priority in international agendas [10]. However, snail control strategies such as the use of molluscicides, non-host competitor snails, and predators are underdeveloped and still evolving [11,12] and knowledge gaps persist regarding drivers of freshwater habitat suitability, IH snail distribution, and associated exposure risks of schistosomiasis transmission, even under climate variability across spatial and temporal scales.

Freshwater habitats and snail distributions in Sub-Saharan Africa are particularly vulnerable to climate variability, including bioclimatic [13,14], geographic (slope, elevation, and proximity to freshwater) [15,16], and environmental (vegetation cover and soil texture) features [13]. Rapid population growth and anthropogenic activities [17], further, drive snail spread. The feature importance of these drivers varies spatially [15,16] and with increasing influence from national to local scales [18]. Furthermore, climate change is expected to reshape snail vector distribution and schistosomiasis transmission dynamics [19,20], but its precise impact remains uncertain [21].

Persistent disease hotspots, such as the Lake Victoria Basin (LVB), exemplify local and small-scale regions with the presence of IH snails and facing significant disease burdens driven by the impact of these drivers [[22], [23], [24]]. While most studies focus on broader, coarse-scale impacts [15,20], there is a critical need for localized, fine-scale analyses to enhance disease surveillance, and informed public health strategies tailored to specific and high-risk local areas. Therefore, this study applies a One Health approach to (i) identify the key drivers and their functional responses, (ii) examine shifts in snail habitat suitability under climate change, and (iii) identify high-risk exposure zones for urogenital and intestinal schistosomiasis, considering the interconnected health of humans, vector snails, and ecosystems.

## 2 Materials and methods

### 2.1 Study area

This study focuses on the Lake Victoria Basin (LVB), spanning Uganda, Kenya, Tanzania, Rwanda, and Burundi, a key ecological and socio-economic region in east Sub-Saharan Africa. Lake Victoria, the world's second-largest freshwater lake by surface area, supports over 42 million people [25]. Population density has risen from 100/km^2^ in 1996 to over 250/km^2^ in 2024 [25]. Its surface elevation is at 1135 m. The residents rely on agriculture, fishing, and domestic water use [25]. Wetlands, rivers, and floodplains provide habitats for *Bulinus* and *Biomphalaria* snails [24,26,27] with *Schistosoma haematobium* and *Schistosoma mansoni* endemic in the region [22,23]. Increasing human pressure, climate change, and ecological drivers of snail vectors increase the vulnerability to disease outbreaks, impacting sustainability and livelihoods [6,28].

### 2.2 Snail occurrence data

**Fig. 1:**
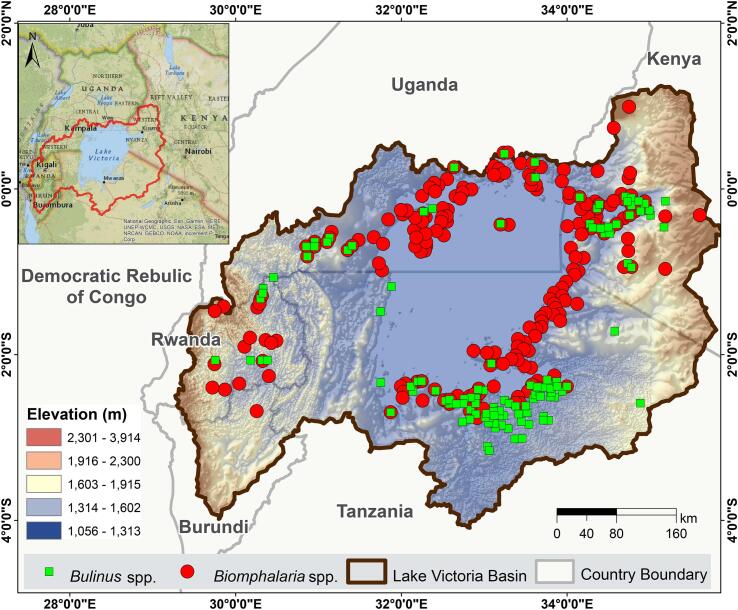
The map showing the occurrence data points for *Bulinus* (green) and *Biomphalaria* (red) in the Lake Victoria basin across the five countries, Uganda, Kenya, Tanzania, Rwanda and Burundi. (For interpretation of the references to colour in this figure legend, the reader is referred to the web version of this article.)

Geographical records of *Biomphalaria* and *Bulinus* species were sourced from GBIF, (https://www.gbif.org/), iNaturalist (https://www.inaturalist.org) with data accessed in 2024 and originally collected over several decades. These were supplemented with data from the literature [15,24,26,27,[29], [30], [31]] and unpublished collections from the Justus Liebig University Giessen, Germany and the Natural History Museum, London, UK (Supplementary S2). The dataset included 324 *Bulinus* and 315 *Biomphalaria* occurrences, providing the most comprehensive species occurrence dataset for the LVB. To minimize over-fitting and spatial autocorrelation, a 5 km buffer was applied using the SDM Toolbox ver. 2.5 in ArcMap 10.8, yielding 210 *Biomphalaria* and 120 *Bulinus* occurrences (Fig. 1). The dataset included various species within each genus such as *B. pfeifferi*, *B. choanomphala*, *B. alexandrina*, and *B. sudanica*, (susceptible to *S. mansoni*) [5] as well as *B. truncatus/tropicus* complex, *B. africanus*, *B. globosus*, *B. ugandae*, *B. nasutus*, *B. productus*, and *B. forskalii* (IH for *S. haematobium*) [5]. However, due to limited data and low occurrences for specific species, the modeling was conducted at the genus level. Although the occurrence records span multiple decades, they were analyzed using environmental predictors representing contemporary conditions (primarily 2023), under the assumption that habitat–climate relationships remain relatively stable over time at the genus level.

### 2.3 Environmental drivers

The drivers including climate (bioclimatic variables), environment (vegetation index, soil texture: silt, sand, and clay content), and topography (elevation, slope, and distance to water bodies) were considered for their biological relevance in controlling snail distribution [15,21,32]. Data sources and resolutions are detailed in Supplementary S1 Table 1. In brief, climate data, including temperature and precipitation, were sourced from the WorldClim V2.1 at a 1 km^2^ resolution to capture annual trends, seasonality, and extreme conditions [33]. Vegetation data, represented by the mean Normalized Difference Vegetation Index (NDVI) from the MODIS (MOD13Q1) product for 2023, were scripted using Google Earth Engine at a 250-m resolution [34]. NDVI reflects land use land cover and human activities that affect water quality parameters like shade, nutrients, and organic matter, which support snail growth and reproduction [35]. Furthermore, different soils vary in their ability (permeability, water retention, and drainage) to leach, adsorb ions, and transport them from the terrestrial environment to nearby freshwater bodies [13]. Soil texture data (silt, sand, clay content) were sourced from the ISRIC Soil Grids database at 250-m resolution, representing the topsoil layer (0–5 cm) [36]. Elevation data (30 m resolution) [37] and slope data derived from the Shuttle Radar Topography Mission (SRTM) using the terra package in R [38] were included. These affect water flow, stability, and suitability for snail populations. Distance to water bodies, a key factor in snail dispersal [29] was calculated via Euclidean distance analysis on water bodies data from DIVA-GIS (https://www.diva-gis.org) in ArcGIS 10.8. All environmental variables were standardized at 1 km resolution using the terra package in R. These variables reflect current ecological conditions and were uniformly applied across all snail occurrence records to ensure consistent model inputs in the absence of temporally resolved environmental layers.

**Table 1:** Proportion of habitat suitability categories (not suitable, marginally suitable, moderately suitable, and highly suitable) under current conditions and future climate scenarios (SSP1–2.6: low emissions, SSP5–8.5: high emissions) for *Bulinus* and *Biompahalaria*.

Suitability Class	Climate Change Scenario					
	Current		SSP1–2.6		SSP5–8.5	
	Area (km^2^)	Coverage (%)	Area (km^2^)	Coverage (%)	Area (km^2^)	Coverage (%)
*Bulinus* spp.						
Not Suitable	136,841	61.51	108,455	48.75	87,584	39.37
Marginally Suitable	44,074	19.81	52,944	23.80	58,414	26.26
Moderately Suitable	19,453	8.74	42,123	18.93	56,471	25.38
Suitable	14,082	6.33	18,825	8.46	19,998	8.99
Highly Suitable	8031	3.61	137	0.06	17	0.01

*Biomphalaria* spp.						
Not Suitable	116,378	52.31	106,424	47.83	101,691	45.71
Marginally Suitable	51,547	23.17	49,937	22.45	58,081	26.11
Moderately Suitable	32,399	14.56	41,680	18.73	46,931	21.09
Suitable	17,063	7.67	23,698	10.65	15,641	7.03
Highly Suitable	5094	2.29	745	0.33	140	0.06

### 2.4 Species distribution model (SDM)

Ensemble SDM was performed in R (v4.4.2) [39] using XGBoost, Random Forest (RF), and MaxEnt, selected to reduce model-specific biases and enhance robustness against multicollinearity and ability to model complex ecological relationships [[40], [41], [42]]. A 10-fold cross-validation (80 % training, 20 % testing) was applied. Pseudo-absence data were generated via the surface-range envelope method, selecting random points outside suitable habitats using a quantile range of 0.025–0.95, maintaining a 1:2 presence-to-absence ratio [43]. Models were run 25 times, and ensemble predictions were weighted by area under the curve (AUC) values. Analyses were conducted in R using dismo, caret, and terra packages, identifying key drivers of *Biomphalaria* and *Bulinus* habitat suitability in the LVB.

### 2.5 Habitat suitability and climate change

Snail distributions were modeled under Shared Socioeconomic Pathways (SSPs) to assess climate change impacts. Two scenarios were considered: SSP1–2.6 (optimistic or low emission scenario) and SSP5–8.5 (pessimistic or high emission scenario), based on CMIP6 projections [44]. Climate data for 2041–2060 were obtained from WorldClim v2.1 using MIROC6 model [33]. Using current NDVI assumes static ecosystem conditions, potentially overlooking shifts driven by environmental changes. In contrast, projecting NDVI under SSP1–2.6 and SSP5–8.5 provides insights into dynamic vegetation responses to varying greenhouse gas trajectories [33] and enhances the accuracy of habitat suitability assessments. NDVI projections under SSP1–2.6 and SSP5–8.5 were generated using a Random Forest algorithm (Supplementary S1 Fig. 1), while soil and topographic variables remained constant. Current models were applied to future datasets to predict habitat suitability under climate change scenarios.

### 2.6 Exposure risk analysis

Schistosomiasis risk under current conditions was assessed by overlaying snail habitat suitability with population density data from the World Gridded Population Dataset (SEDAC) at 1 km resolution (https://earthdata.nasa.gov/centers/sedac-daac). Population density was classified into five categories: very low (0–1 persons/km^2^), low (>1–10), medium (>10–100), high (100−1000), and very high (>1000). Habitat suitability, derived from the ensemble model, was similarly classified into five categories using the equal interval method [17]. Risk maps were generated by multiplying habitat suitability and population density rasters using ArcGIS 10.8. The resulting maps identified regions where human populations overlap with suitable snail habitats, highlighting schistosomiasis transmission hotspots.

There are no future projections for population density tailored for the LVB. However, global gridded datasets from Zhuang et al. [45] were adapted for the region. Future schistosomiasis risk was assessed by overlaying these with projected habitat suitability maps, using the same approach as for current conditions.

## 3 Results

### 3.1 Model performance and feature contributions

**Fig. 2:**
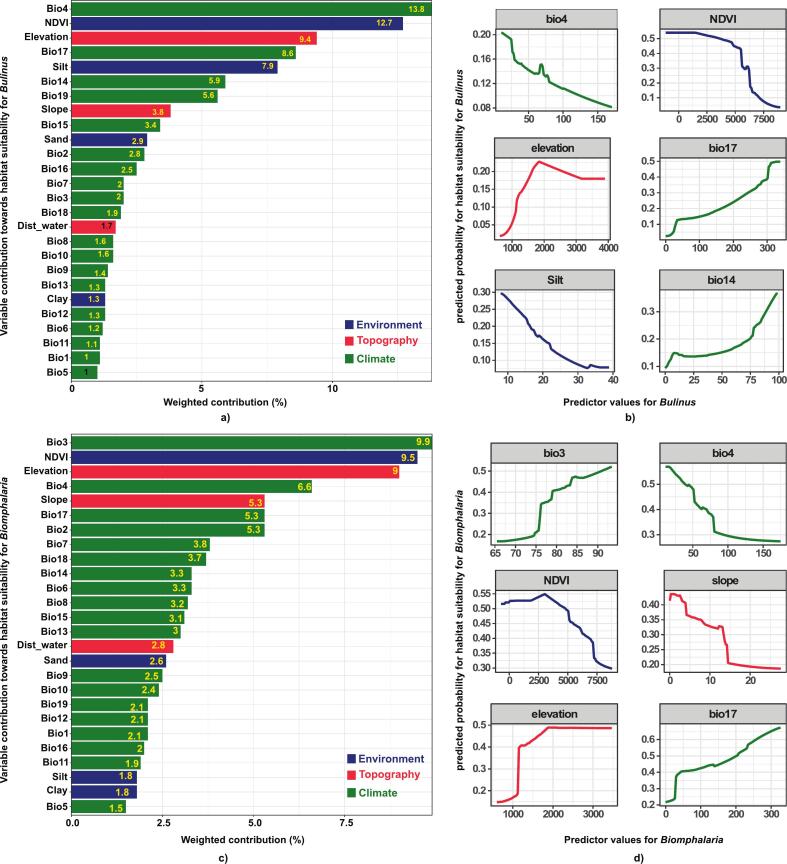
Variable contributions to habitat suitability for (a) *Bulinus*, with response curves illustrating the effects of the top six predictors on its occurrences. Similarly, (c) shows the variable contributions for *Biomphalaria*, and *(d)* depicts the response curves for its occurrences. Abbreviations: Bio1: Annual Mean Temperature, Bio2: Mean Diurnal Range, Bio3: Isothermality, Bio4: Temperature Seasonality, Bio5: Maximum Temperature of Warmest Month, Bio6: Minimum Temperature of Coldest Month, Bio7: Temperature Annual Range, Bio8: Mean Temperature of Wettest Quarter, Bio9: Mean Temperature of Driest Quarter, Bio10: Mean Temperature of Warmest Quarter, Bio11: Mean Temperature of Coldest Quarter, Bio12: Annual Precipitation, Bio13: Precipitation of Wettest Month, Bio14: Precipitation of Driest Month, Bio15: Precipitation Seasonality, Bio16: Precipitation of Wettest Quarter, Bio17: Precipitation of Driest Quarter, Bio18: Precipitation of Warmest Quarter, Bio19: Precipitation of Coldest Quarter, Dist_water: Distance to Water Bodies. Refer to Supplementary S1, Fig. 2, and Fig. 3 for the predicted occurrence probabilities of *Bulinus* and *Biomphalaria* across all environmental, topographic and climatic variables.

The geographical range of *Bulinus* and *Biomphalaria* genera in the LVB spans 1° N to 4° S latitude and 31° E to 36° E longitude, predominantly occurring at elevations between 1135 m (i.e., the lake's surface elevation) and 2542 m for *Bulinus*, 1135 m and 2083 for *Biomphalaria* (Fig. 1). Statistical analysis provided the minimum, maximum, and standard deviation values for each variable within the basin. The results revealed slight variations in the variable ranges for both genera, suggesting differences in their environmental niche requirements (Supplementary S1 Table 2). All models showed strong predictive accuracy, with AUC values for *Bulinus* of 0.959 (RF), 0.940 (XGBoost), and 0.956 (MaxEnt), and for *Biomphalaria*, 0.941 (RF), 0.924 (XGBoost), and 0.943 (MaxEnt), respectively. These results highlight the robustness of the ensemble model to accurately represent the true distributions of IH snail genera (Fig. 1), and can provide dependable predictions. Ensemble models revealed that climate-related variables were the most significant predictors of snail occurrences, surpassing the influence of topographic and environmental factors (Fig. 2). Predicted habitat suitability probabilities exhibited non-linear trends, emphasizing subtle distinctions in the freshwater habitat preferences of the two genera. For *Bulinus*, the six most critical predictors were temperature seasonality (Bio4), precipitation during the driest quarter (Bio17) and driest month (Bio14), NDVI, silt content, and elevation (Fig. 2a). The likelihood of *Bulinus* occurrence increased with higher Bio17, Bio14, and elevation (<2000 m) but decreased with rising NDVI, silt content, and Bio4 (Fig. 2b). In contrast, *Biomphalaria* distribution was predominantly influenced by isothermality (Bio3), temperature seasonality (Bio4), precipitation during the driest quarter (Bio17), NDVI, elevation, and slope (Fig. 2c). Predicted suitability for *Biomphalaria* increased in areas with higher Bio13, and Bio17, reaching a peak at an approximate elevation of 2000 m. However, suitability declined with higher NDVI, slope, and Bio4 values (Fig. 2d).

### 3.2 Distinct areas of habitat suitability distribution

**Fig. 3:**
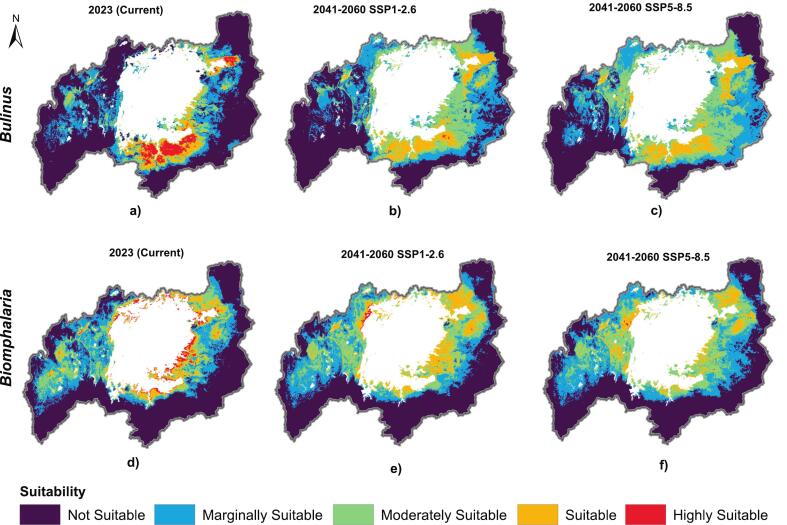
Habitat suitability maps for *Bulinus* (top panels) and *Biomphalaria* (bottom panels) genera in the Lake Victoria Basin under current conditions (first column: a, d) and projected future climate scenarios based on Shared Socioeconomic Pathways (SSPs) from 2041 to 2060. The middle column (b, e) represents a low-emission scenario (SSP1–2.6), while the last column (c, f) depicts a high-emission scenario (SSP5–8.5). See supplementary S1 Fig. 4 for unsuitable and suitable areas of habitat suitability for both genera.

**Fig. 4:**
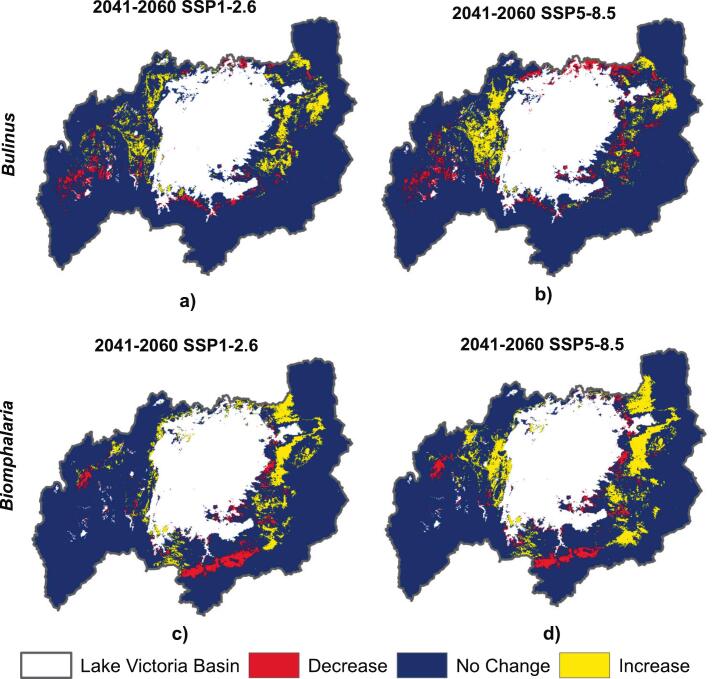
Changes in habitat suitability for *Bulinus* (top panels) and *Biomphalaria* (bottom panels) genera between current conditions and future projections under Shared Socioeconomic Pathways (SSPs) for 2041–2060. (a, c) correspond to the low-emission scenario (SSP1–2.6), while (b, d) represent the high-emission scenario (SSP5–8.5). Areas in red indicate a decrease in habitat suitability, yellow indicates an increase, and blue denotes no change. See the supplementary S1 Fig. 5 for graphical representation of the quantified areas of habitat suitability. (For interpretation of the references to colour in this figure legend, the reader is referred to the web version of this article.)

The predicted habitat suitability area for *Biomphalaria* and *Bulinus* reveals distinct distribution patterns, with both genera concentrated predominantly in the eastern LVB, while the western region shows a slightly higher presence of *Biomphalaria* compared to *Bulinus* (Fig. 3). Habitat suitability is highest near lake shores and decreases with increasing distance from the lake. The current LVB distribution covers 222,481 km^2^, with *Biomphalaria* occupying a significantly larger suitable area of 37,953 km^2^ (17.06 % of the total area), particularly in western Kenya, central Uganda, and northern Tanzania, some parts of Rwanda and Burundi (Fig. 3d; Supplementary S1 Table 3). In contrast, *Bulinus* has a smaller suitable area of 31,986 km^2^ (14.38 % of the total area), mainly distributed across parts of northern Tanzania, western Kenya, and eastern and central Uganda (Fig. 3a; Supplementary S1 Table 3). The small-scale shifts in habitat suitability under future climate scenarios indicate that both genera respond consistently to projected climate change (Fig. 3b, e, c, f). Overall, both genera exhibit an increasing trend in suitable regions, though *Biomphalaria* maintains a higher suitability compared to *Bulinus*. However, some areas will increase in suitable area coverage, others will experience a decline, and some remain unchanged (Fig. 4a-d). Specifically, *Biomphalaria* suitability is projected to increase from 37,953 km^2^ in 2023 to 47,169 km^2^ (21.20 % of the total area) under the SSP1–2.6 scenario and to 41,290 km^2^ (18.56 % of the total area) under the SSP5–8.5 scenario by 2060. For *Bulinus*, suitable area rises from 31,986 km^2^ in 2023 to 40,551 km^2^ (18.23 % of the total area) under SSP1–2.6 and to 47,439 km^2^ (21.32 % of the total area) under SSP5–8.5 by 2060 (Supplementary S1, Table 4).

Although suitable areas for both *Bulinus* and *Biomphalaria* are projected to expand under future climate change scenarios, further classification indicates a significant reduction in highly suitable areas. Specifically, for *Bulinus*, the proportion of highly suitable habitat is expected to decrease from 3.61 % under current conditions to 0.06 % under SSP1–2.6 and further to 0.01 % under SSP5–8.5. Similarly, for *Biomphalaria*, highly suitable areas are projected to decline from 2.29 % currently to 0.33 % under SSP1–2.6, and to 0.06 % under SSP5–8.5. These findings highlight the potential for significant habitat shifts and the need for targeted conservation and public health strategies (Table 1). The maps depicting areas classified as not suitable, marginally suitable, moderately suitable, and highly suitable within the basin are provided in Supplementary S1 Fig. 6. A graphical representation of the quantified areas of habitat suitability across different classifications for both genera is also available (Supplementary S1 Fig. 7).

### 3.3 Future schistosomiasis exposure risk

**Fig. 5:**
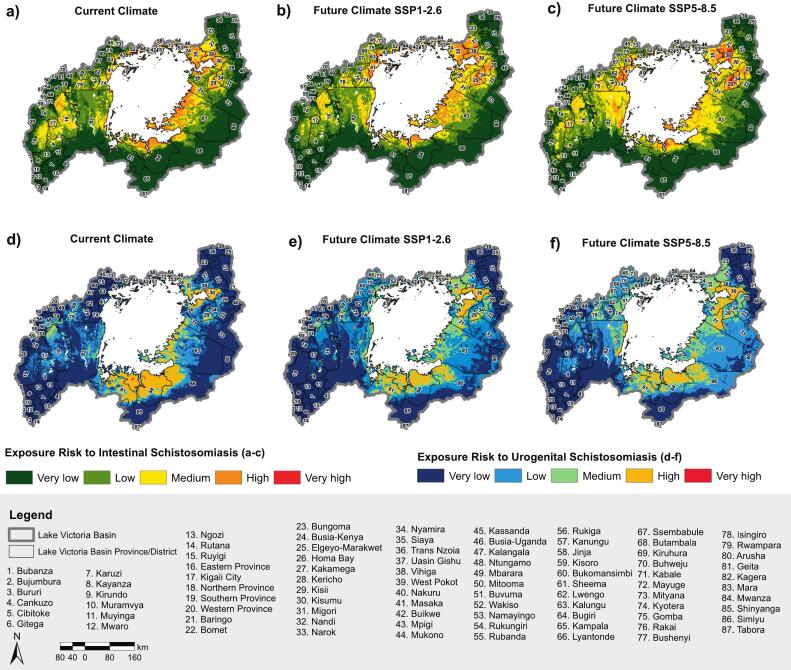
Geographical distribution of potential exposure risk to urogenital schistosomiasis (a-c) and intestinal schistosomiasis (d-f) due to *Bulinus* and *Biomphalaria* habitat suitability and population exposure under current and future climate conditions in the Lake Victoria Basin. The exposure risk for each geographical region 1–87 depicted in Fig. 5 is given in Supplementary S 3.

The current and future risk distributions of urogenital and intestinal schistosomiasis infections from very low to very high were mapped and specific patterns in certain districts, provinces, and sub-counties within the basin were identified (Fig. 5a-f; Supplementary S1 Table 4; Supplementary S3).

The current exposure risk of intestinal schistosomiasis is concentrated around Lake Victoria, particularly in the southern and eastern regions, with notable hotspots in Kisumu (30), Siaya (35), Vihiga (38), Mara (83), and Mwanza (84), along with isolated cases in Eastern Province (16 and 17) (Fig. 5a). Under future climate change and population growth scenarios, the spatial extent of intestinal schistosomiasis risk is projected to expand, with regional variations in intensity. Very low-risk areas of intestinal schistosomiasis are expected to decrease from 52.70 % to 48.81 % (SSP1–2.6) and 45.09 % (SSP5–8.5), while medium-risk zones increase from 13.3 % to 15.91 % and 21.97 %, respectively. High-risk areas initially rise under SSP1–2.6 (9.08 %) but decline under SSP5–8.5 (6.41 %), whereas very high-risk areas nearly disappear under SSP1–2.6 (0.01), indicating a shift in the disease burden rather than a uniform decline but increase to 1.11 % under SSP5–8.5. Emerging hotspots are anticipated in Kericho (28), Nyamira (34), Mara (83), and western areas such as Masaka (41).

In contrast, urogenital schistosomiasis is more prevalent in the eastern and southern regions, with major hotspots in Kisumu (30), Geita (81), and Mwanza (84) (Fig. 5d). Similarly, for future projections of urogenital schistosomiasis, very low-risk areas are projected to decline from 62.01 % to 49.99 % (SSP1–2.6) and 41.06 % (SSP5–8.5), with medium-risk zones nearly double from 7.93 % to 14.48 % (SSP1–2.6) and more than double to 17.67 % (SSP5–8.5), respectively. High-risk areas relatively reduce from 9.12 % to 6.73 % (SSP1–2.6) and 6.89 % (SSP5–8.5), while very high-risk zones currently minimal (0.09 %) are projected to disappear entirely under both scenarios, suggesting a potential reduction in extreme transmission hotspots. This form of schistosomiasis is expected to broaden in spatial distribution, with new areas of concern emerging around the lake basin, particularly in Arusha (80), Simiyu (86), Bukomansimbi (60), and Kassanda (45), where low to high risk is projected. These findings emphasize the spatial variability and localized nature of schistosomiasis risk across the basin.

## 4 Discussion

Freshwater habitats in the LVB are highly vulnerable to climatic, topographic, and environmental changes due to their physical isolation and fragmentation, often within landscapes that are heavily exploited and modified by human activities [25]. These changes influence snail habitat suitability and schistosomiasis risk across spatial scales [18,46]. Alterations in freshwater habitats have been linked to rising schistosomiasis cases [21,23]. This study applies One Health principles by integrating environmental and socio-ecological drivers to assess shifts in *Biomphalaria* and *Bulinus* habitat suitability under climate change and map schistosomiasis risk zones.

Our findings confirm that climate-related variables (temperature and precipitation) are the dominant predictors of snail habitat suitability, aligning with broader-scale studies [13,20,32,46]. Differences in predictors between *Biomphalaria* and *Bulinus* reflect their distinct dispersal abilities, drought resistance, and environmental adaptations [5,16,17], yet the ecological traits of each genus provide further insights. In the LVB, *Biomphalaria* is generally more sensitive to temperature isothermality, and precipitation of driest quarter, indicating an adaptation to specific climatic conditions [5,13]. *Bulinus* is more reliant on temporary water bodies formed during specific precipitation patterns, such as the coldest quarter or driest month, which aligns with their drought resistance and ability to thrive in environments with intermittent water availability with long-term desiccation, making their dispersal more influenced by short-term seasonal water availability than prolonged retention [5,13,32]. Elevation influences both genera, which thrive below 2000 m, consistent with previous findings on altitude limits [16,29,47]. It regulates water flow dynamics, contributing to habitat stability or instability for snails, while flatter terrain facilitates water accumulation, creating favorable conditions for vector snail populations. Nonetheless, increasing temperature seasonality and NDVI limit both genera, suggesting thermal stress and ecological constraints limiting their spread in such environments [13,20]. High-temperature seasonality disrupts physiological processes, reducing reproductive success and survival rates [13]. High NDVI, indicating dense vegetation, may create suboptimal conditions through shading, carbon dioxide emission from decomposing submerged vegetation, oxygen depletion, and increased evapotranspiration, reflecting unfavorable habitat conditions [15,17]. *Bulinus* is further constrained by high silt content, while *Biomphalaria* is influenced by slope, reflecting ecological niche variations that may affect their coexistence or competition within the basin. The negative correlation with silt is also observed in more recent studies [17,19]. High silt levels increase water retention and reduce nutrient losses via leaching into freshwater habitats, limiting essential nutrients like nitrates and calcium, which disrupts snail life cycles and survival [48]. Steep slopes further restrict *Biomphalaria* by increasing stream velocity, reducing organic matter accumulation, and destabilizing habitats, ultimately constraining its distribution [13,32]. These habitat predictors highlight the adaptability of snails while underscoring their vulnerability to climate-driven environmental changes.

The genus-specific responses to climate change account for the distinct geographic patterns of habitat suitability observed in this study. Our predictions indicate concentrated habitat suitability near lake shores and urban centers for both genera. Currently, the increasing schistosomiasis cases [22,23] and seasonal disease re-emergence [19] in the region raise concerns about transmission risks. The presence of infected snail vectors [24,27] further exacerbates exposure in densely populated and industrialized areas [25], where specific abiotic factors and sewage conditions facilitate the persistence of snail populations [49]. Furthermore, human reliance (fishing, agriculture, tourism and recreation) on LVB water resources, coupled with poor sanitation, increases schistosomiasis risk [28]. Moreover, Urogenital schistosomiasis, prevalent in LVB [50], is associated with increased vulnerability to co-infections like HIV/AIDS, particularly in fishing communities where frequent water exposure and socio-behavioral factors, including alcohol consumption and increased rates of sexual activity between local populations and sex workers, elevate risks [51]. This dual burden poses significant health and socio-economic challenges across LVB countries [25,28].

Climate projections indicate a substantial increase in suitable snail habitats and infection risks in LVB, with *Biomphalaria* experiencing greater expansion in suitability and associated risks than *Bulinus*. Increased precipitation and higher temperature isothermality, reflecting more uniform/stable temperatures between day and night, will foster conditions favorable for snail colonization and schistosomiasis transmission [27]. Stable regions will sustain existing populations, while transitional areas may see local extinctions as conditions exceed tolerance limits [46]. Additionally, transient favorable conditions during otherwise unfavorable periods could temporarily enable the re-establishment of snail populations, heightening habitat suitability and associated transmission risks [20,21]. Therefore, expansion zones and the associated risks are expected to vary for both genera, however, some zones like Kisumu may emerge as dual hotspots for infections due to overlapping habitat suitability. These findings align with broader studies indicating localized risk zones rather than uniform distributions [13,19,20]. Conversely, reduced suitability for *Biomphalaria* in Rukiga and *Bulinus* in Eastern Province contributes to lower future infection risks, highlighting the complex interplay of climatic and environmental stressors [13]. Localized reductions may lower transmission risks, but expansions elsewhere could maintain the overall disease burden.

## 5 Conclusion

This study underscores the significant impact of climate change on *Biomphalaria* and *Bulinus* habitat suitability in the LVB. Climate, topographic, and environmental factors, particularly temperature seasonality, precipitation, NDVI, and elevation shape snail distributions, with both genera concentrated near lake shores. Future scenarios predict an expansion of suitable habitats and increased risks of intestinal and urogenital schistosomiasis. These findings emphasize the need for localized disease surveillance, snail control, and climate-adaptive strategies. The study supports One Health approaches for public health interventions. However, due to the lack of basin-specific demographic, snail infection, and water contact data, as well as our primary focus on environmental suitability modeling, our projections of future risk rely on generalized assumptions. Future research should incorporate these critical datasets, along with demographic projections, to enhance species distribution models and improve the accuracy and effectiveness of schistosomiasis control strategies.

## CRediT authorship contribution statement

**Zadoki Tabo:** Writing – original draft, Visualization, Resources, Methodology, Formal analysis, Data curation, Conceptualization. **Rapheal Wangalwa:** Writing – review & editing, Visualization, Software, Methodology, Formal analysis, Data curation. **Marcellin Rwibutso:** Writing – review & editing, Visualization, Resources. **Lutz Breuer:** Writing – review & editing, Supervision, Project administration, Investigation, Funding acquisition, Conceptualization. **Christian Albrecht:** Writing – review & editing, Supervision, Resources, Investigation, Conceptualization.

## Funding statement

This study was funded by a seed grant from the Center for International Development and Environmental Research (ZEU) at Justus Liebig University Giessen, Germany.

## Declaration of competing interest

The authors declare that they have no conflicts of interest.

## Data Availability

All data supporting the reported results are included within the article and supplementary files. Supplementary File S1 contains additional figures and tables illustrating the estimation of the dynamic vegetation index, key features for both genera and maps and tables depicting habitat suitability and potential risk zones. Supplementary File S2 contains the correlated data used in this study. The algorithms for data analysis are available at this GitHub repository.

## Appendix A Supplementary data

Supplementary material 1 Image 1

Supplementary material 2 Image 2

Supplementary material 3 Image 3
