# Supplementary material for: Future climate and demographic changes will almost double the risk of schistosomiasis transmission in the Lake Victoria Basin
Source: One Health. 2025 Jul 18;21:101148. doi: 10.1016/j.onehlt.2025.101148 (PMC12305727; doi:10.1016/j.onehlt.2025.101148)
Supplement: Supplementary file 1 — Supplementary material 1 [file mmc1.docx]

**Supplementary S1**

**For Future climate and demographic changes will almost double the risk of schistosomiasis transmission in the Lake Victoria Basin**

**Zadoki Tabo^a,b*^, Rapheal Wangalwa^d^, Marcellin Rwibutso^c^, Lutz Breuer^a,b^ & Christian Albrecht^c,d^**

^a^Centre for International Development and Environmental Research (ZEU), Justus Liebig University Giessen, Senckenbergstrasse 3, 35390 Giessen, Germany

^b^Institute for Landscape Ecology and Resources Management (ILR), Research Centre for Biosystems, Land Use and Nutrition (iFZ), Justus Liebig University Giessen, Heinrich-Buff-Ring 26, 35392 Giessen, Germany.

^c^Department of Animal Ecology and Systematics, Justus Liebig University Giessen, Heinrich-Buff-Ring 26 (iFZ), 35392 Giessen, Germany.

^d^Department of Biology, Mbarara University of Science and Technology (MUST), P. O. Box 1410, Mbarara, Uganda.

**^*^**Corresponding Author

E-mail: [Tabo.Zadoki@umwelt.uni-giessen.de](mailto:Tabo.Zadoki@umwelt.uni-giessen.de) (ZT)

**Material and methods**

**Environmental and demographical data**

Table 1 summarizes the environmental and demographic drivers used for habitat suitability modeling of snail intermediate hosts of schistosomiasis in the Lake Victoria Basin. The table includes data sources, spatial and temporal resolutions, and thematic categories such as climate, vegetation, soil properties, and topography. **Table 2** presents the statistical summary of environmental, topographic, and climatic variables, offering insights into their distributions and descriptive measures.

**Supplementary S1 Table 1.** Summary of environmental and demographic variables used in habitat suitability modeling, including climate (bioclimatic variables), vegetation (e.g., NDVI), soil texture (silt, sand, clay content), and topographic features (elevation, slope, and proximity to water bodies)

| **Data category** | **Variables** | **Spatial Resolution** | **Year** | **Sources** |
| --- | --- | --- | --- | --- |
| Climate | Bioclimatic Variables | 30 arc-seconds (~1 km) | 1970-2000  2041-2060 | WorldClim ver.2.1 (<https://www.worldclim.org/>) |
| Environment | Vegetation Index (NDVI) | 250m | 2023 | MODIS products in Google Earth Engine (<https://code.earthengine.google.com>) |
|  | Silt, Sand, Clay | 250m | 1960-2015 | ISRIC Soil Grids (<https://data.isric.org/>) |
| Topography | Elevation | 30m | 2000 | SRTM-DEM in GEE (<https://code.earthengine.google.com> ) |
|  | Slope | 30m | 2000 | Derived from SRTM-DEM in GEE (<https://code.earthengine.google.com> ) |
|  | Inland Water (Distance to Water Body) | Country | 2007 | DIVA-GIS (<https://diva-gis.org/data.html> ) |
| Demographical | Current Population | 30 arc-seconds (~1 km) | 2020 | CIESIN-ESDIS (<https://search.earthdata.nasa.gov/>) |
|  | Population projections | 30 arc-seconds (~1 km) | 2020-2100 | Zhuang et al., 2024 ([https://doi.org/10.1016/j.scitotenv.2024.176867](https://doi.org/10.1016/j.scitotenv.2024.176867)) |

**Supplementary S1 Table 2:** Summary statistics of all variables across habitats of *Bulinus* and *Biompahalaria* snails in the Victoria basin. Blue=environmental variables, red=topographic variables, green=climatic variables

| **Variables** | ***Bulinus*** | | | | ***Biomphalaria*** | | | |
| --- | --- | --- | --- | --- | --- | --- | --- | --- |
|  | **Mean** | **SD** | **Min** | **Max** | **Mean** | **SD** | **Min** | **Max** |
| Bio1: Annual Mean Temperature (°C) | 22.13 | 1.20 | 14.20 | 23.30 | 21.55 | 1.46 | 16.42 | 23.42 |
| Bio2: Mean Diurnal Range (°C) | 11.42 | 0.71 | 8.63 | 12.63 | 10.27 | 1.53 | 7.42 | 13.31 |
| Bio3: Isothermality | 78.83 | 4.63 | 72.39 | 88.81 | 83.19 | 2.71 | 75.05 | 91.90 |
| Bio4: Temperature Seasonality (°C) | 59.01 | 16.34 | 24.34 | 102.12 | 48.86 | 14.40 | 24.40 | 75.00 |
| Bio5: Max Temperature of Warmest Month (°C) | 29.29 | 1.71 | 19.90 | 31.40 | 27.75 | 1.81 | 22.50 | 31.40 |
| Bio6: Min Temperature of Coldest Month (°C) | 14.72 | 0.92 | 8.80 | 16.60 | 15.41 | 1.64 | 10.00 | 17.30 |
| Bio7: Temperature Annual Range (°C) | 14.57 | 1.52 | 10.40 | 16.50 | 12.35 | 1.81 | 9.00 | 15.90 |
| Bio8: Mean Temperature of Wettest Quarter (°C) | 22.28 | 1.24 | 13.95 | 23.35 | 21.72 | 1.58 | 16.32 | 23.50 |
| Bio9: Mean Temperature of Driest Quarter (°C) | 21.65 | 1.08 | 14.25 | 23.67 | 21.23 | 1.45 | 16.20 | 23.75 |
| Bio10: Mean Temperature of Warmest Quarter (°C) | 22.85 | 1.35 | 14.50 | 24.20 | 22.11 | 1.59 | 16.68 | 23.93 |
| Bio11: Mean Temperature of Coldest Quarter (°C) | 21.41 | 1.05 | 13.80 | 22.40 | 20.93 | 1.29 | 16.02 | 22.87 |
| Bio12: Annual Precipitation (mm) | 971.28 | 169.87 | 767.00 | 1923.00 | 1202.08 | 305.63 | 767.00 | 2099.00 |
| Bio13: Precipitation of Wettest Month (mm) | 160.27 | 27.81 | 115.00 | 339.00 | 194.02 | 42.29 | 115.00 | 335.00 |
| Bio14: Precipitation of Driest Month (mm) | 12.77 | 16.09 | 2.00 | 80.00 | 35.04 | 27.60 | 3.00 | 98.00 |
| Bio15: Precipitation Seasonality (%) | 63.64 | 11.05 | 35.50 | 76.50 | 51.26 | 8.43 | 34.94 | 70.96 |
| Bio16: Precipitation of Wettest Quarter (mm) | 401.87 | 68.71 | 275.00 | 826.00 | 485.78 | 127.92 | 275.00 | 902.00 |
| Bio17: Precipitation of Driest Quarter (mm) | 60.53 | 62.91 | 13.00 | 295.00 | 136.02 | 83.57 | 29.00 | 299.00 |
| Bio18: Precipitation of Warmest Quarter (mm) | 223.16 | 49.83 | 137.00 | 688.00 | 276.81 | 82.38 | 148.00 | 513.00 |
| Bio19: Precipitation of Coldest Quarter (mm) | 87.92 | 104.33 | 13.00 | 523.00 | 192.43 | 98.44 | 33.00 | 458.00 |
| Clay Content (%) | 25.07 | 9.72 | 9.14 | 43.33 | 33.08 | 6.48 | 14.89 | 51.14 |
| Silt Content (%) | 16.38 | 5.43 | 8.82 | 33.90 | 22.11 | 4.12 | 9.56 | 30.96 |
| Sand Content (%) | 58.56 | 14.49 | 31.66 | 80.54 | 44.83 | 8.80 | 28.90 | 73.12 |
| NDVI | 5176.90 | 932.86 | 2285.02 | 7578.22 | 4870.53 | 1530.42 | 1274.86 | 8006.96 |
| Elevation (m) | 1259.39 | 170.45 | 1135.29 | 2542.30 | 1267.62 | 211.83 | 1135.29 | 2083.14 |
| Slope (°) | 3.19 | 2.70 | 0.78 | 19.10 | 4.06 | 2.80 | 0.79 | 13.88 |
| DW: Distance from Waterbody (m) | 3161.56 | 2541.40 | 0.00 | 12365.05 | 1421.91 | 1375.28 | 0.00 | 9394.71 |

**NDVI Projection**

Future NDVI was projected under two climate change scenarios, SSP1-2.6 (low-emission) and SSP5-8.5 (high-emission), using bioclimatic variables for the period 2041-2060 derived from the MIROC6 Global Climate Model. All raster datasets were resampled to a spatial resolution of 1 km using bilinear interpolation with the terra package to ensure consistency across layers. To model the relationship between NDVI and environmental predictors, a Random Forest (RF) machine learning algorithm [1] was employed. A total of 5,000 random points were generated across the study area to extract response (NDVI) and predictor (bioclimatic and topographic) variables. This sampling approach captured spatial variability while maintaining computational efficiency. Model training was performed using the caret package in R [2], with 10-fold cross-validation used for hyperparameter tuning and performance evaluation. The final RF model used 500 trees (ntree = 500), and the optimal number of variables considered at each split (mtry) was determined via grid search. Model performance was assessed using Root Mean Square Error (RMSE) and Mean Absolute Error (MAE), yielding values of 0.060 and 0.042, respectively, indicating strong predictive performance [3]. The trained RF model was then applied to the future climate scenarios to generate NDVI projections for each SSP, and spatial maps were produced and presented in Fig 1.

**
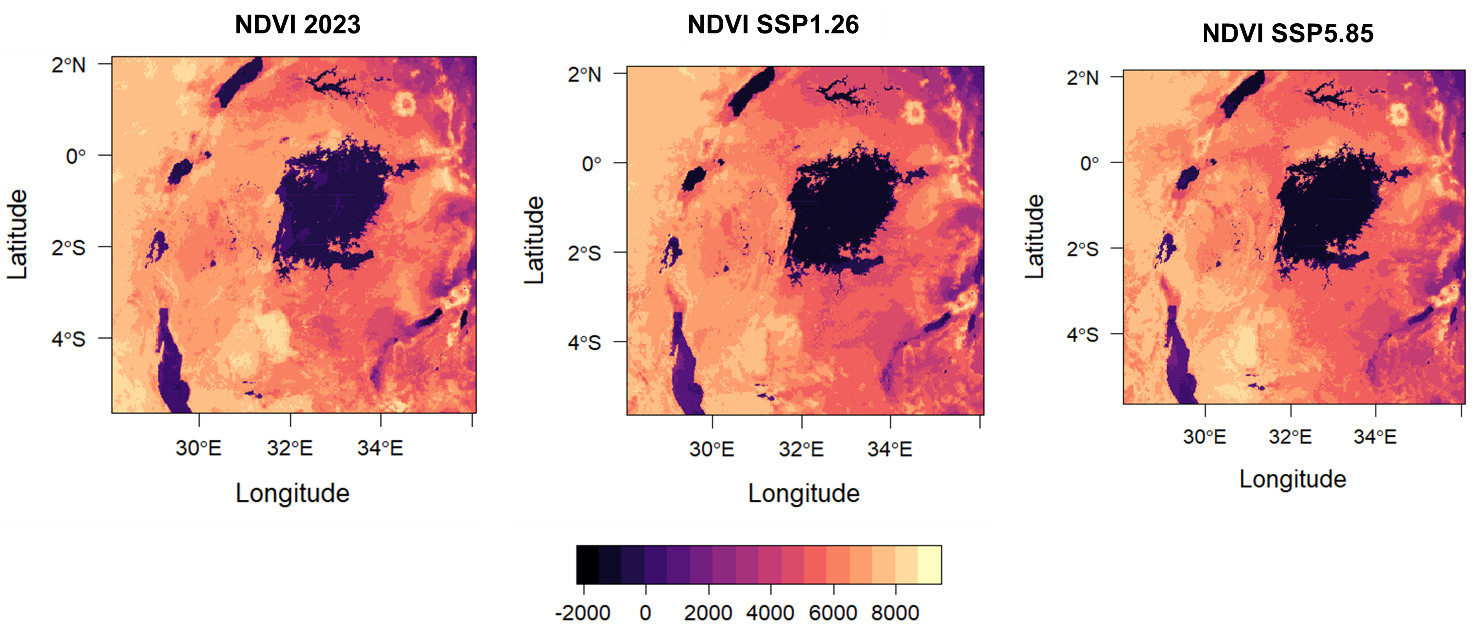
**

**Supplementary S1 Fig. 1.** Projected NDVI for the Lake Victoria Basin under current conditions (2023), and future low-emission (SSP1-2.6) and high-emission (SSP5-8.5) scenarios for the period 2041–2060.

**
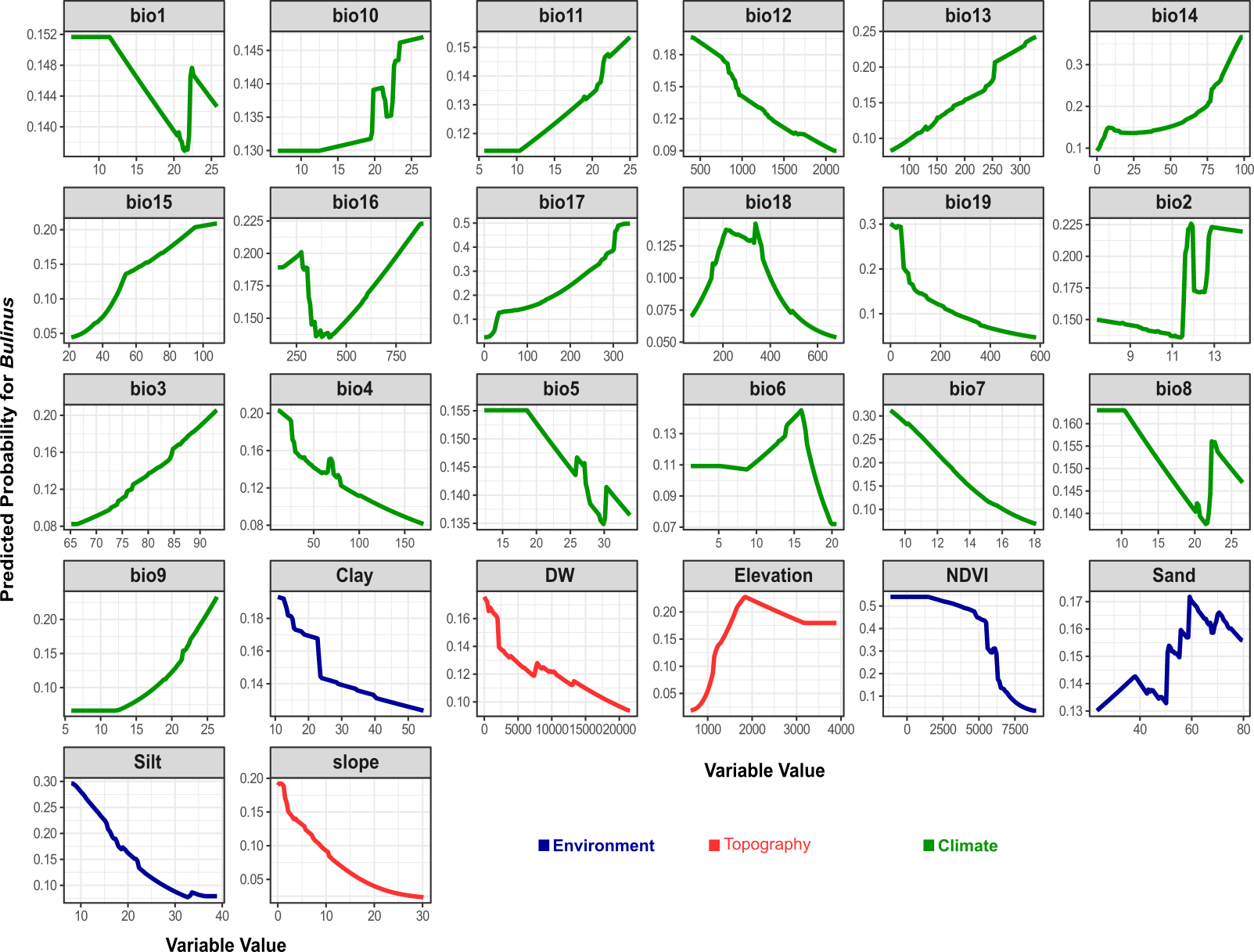
 Supplementary S1 Fig. 2**. Predicted occurrence probabilities of *Bulinus* across all environmental, topographical and climatic variables. Abbreviations: Bio1: Annual Mean Temperature, Bio2: Mean Diurnal Range, Bio3: Isothermality, Bio4: Temperature Seasonality, Bio5: Maximum Temperature of Warmest Month, Bio6: Minimum Temperature of Coldest Month, Bio7: Temperature Annual Range, Bio8: Mean Temperature of Wettest Quarter, Bio9: Mean Temperature of Driest Quarter, Bio10: Mean Temperature of Warmest Quarter, Bio11: Mean Temperature of Coldest Quarter, Bio12: Annual Precipitation, Bio13: Precipitation of Wettest Month, Bio14: Precipitation of Driest Month, Bio15: Precipitation Seasonality, Bio16: Precipitation of Wettest Quarter, Bio17: Precipitation of Driest Quarter, Bio18: Precipitation of Warmest Quarter, Bio19: Precipitation of Coldest Quarter, DW: Distance to Water Bodies


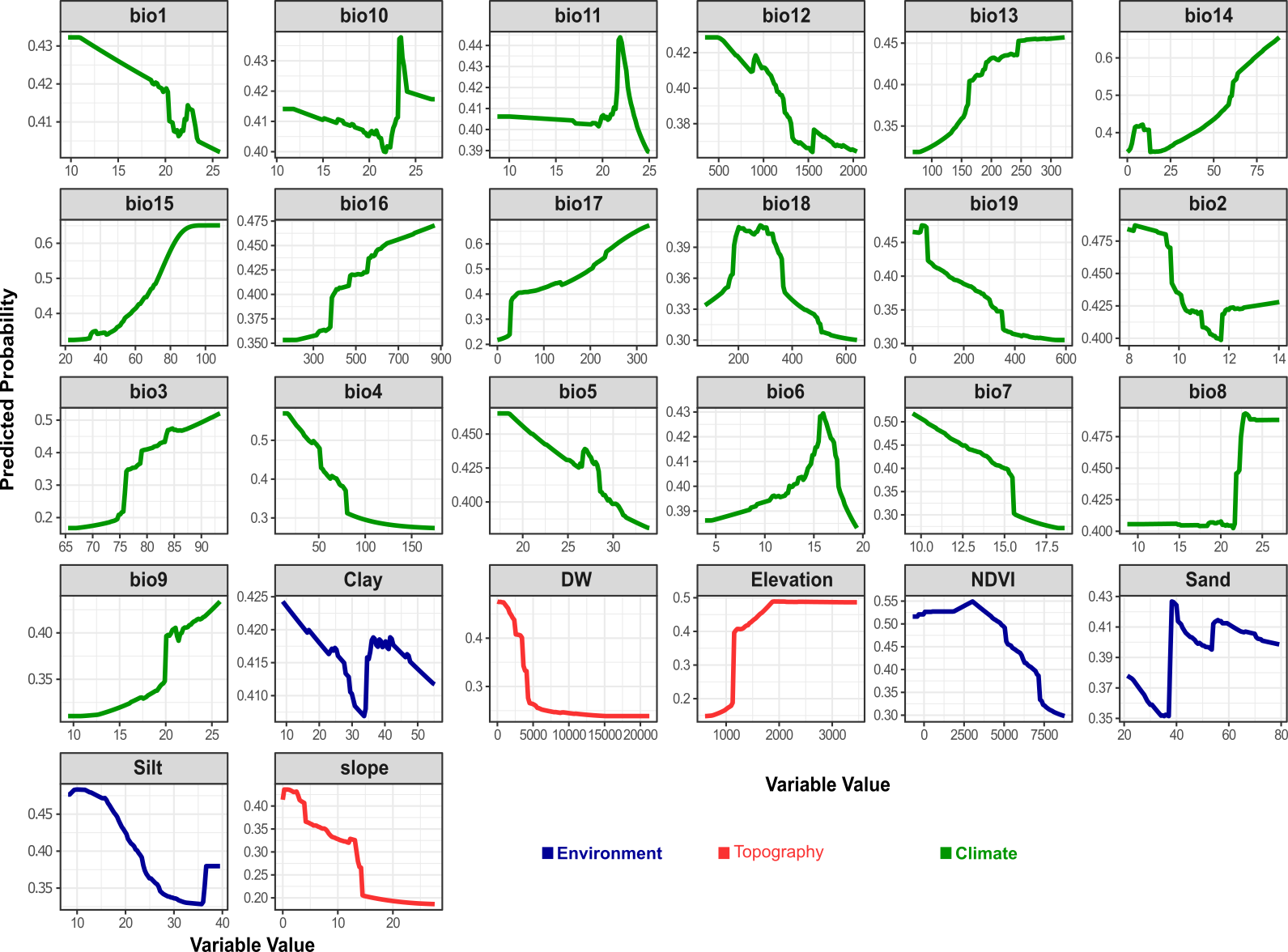


**Supplementary S1 Fig. 3.** Predicted occurrence probabilities of *Biomphalaria* across all environmental, topographical and climatic variables. Abbreviations: Bio1: Annual Mean Temperature, Bio2: Mean Diurnal Range, Bio3: Isothermality, Bio4: Temperature Seasonality, Bio5: Maximum Temperature of Warmest Month, Bio6: Minimum Temperature of Coldest Month, Bio7: Temperature Annual Range, Bio8: Mean Temperature of Wettest Quarter, Bio9: Mean Temperature of Driest Quarter, Bio10: Mean Temperature of Warmest Quarter, Bio11: Mean Temperature of Coldest Quarter, Bio12: Annual Precipitation, Bio13: Precipitation of Wettest Month, Bio14: Precipitation of Driest Month, Bio15: Precipitation Seasonality, Bio16: Precipitation of Wettest Quarter, Bio17: Precipitation of Driest Quarter, Bio18: Precipitation of Warmest Quarter, Bio19: Precipitation of Coldest Quarter, Dist_water: Distance to Water Bodies


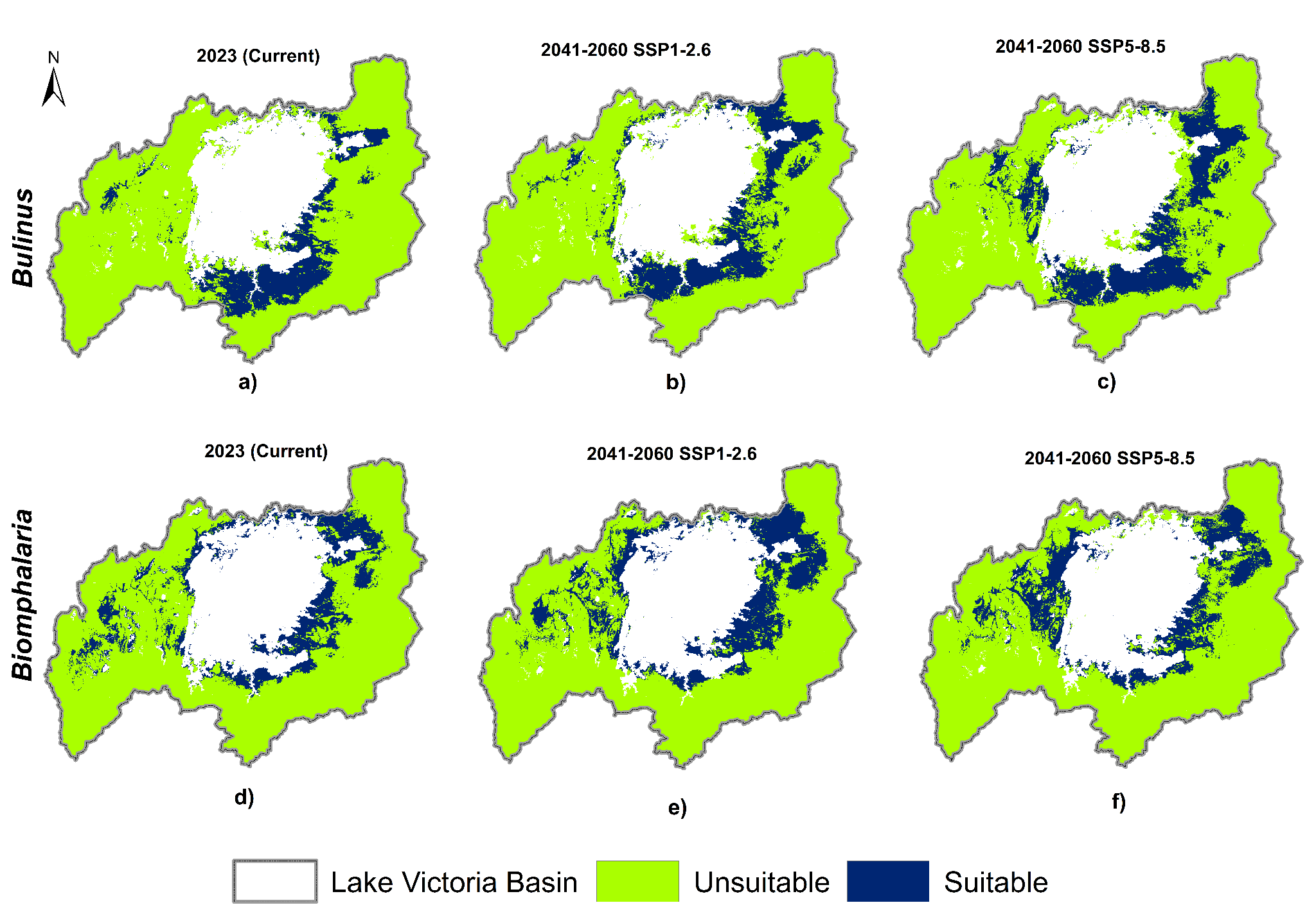


**Supplementary S1 Fig. 4:** Habitat suitability maps classified as unsuitable and suitable for *Bulinus* (top panels) and *Biomphalaria* (bottom panels) genera in the Lake Victoria Basin under current conditions (first column: a, d) and projected future climate scenarios based on Shared Socioeconomic Pathways (SSPs) from 2041 to 2060. The middle column (b, e) represents a low-emission scenario (SSP1-2.6), while the last column (c, f) depicts a high-emission scenario (SSP5-8.5).


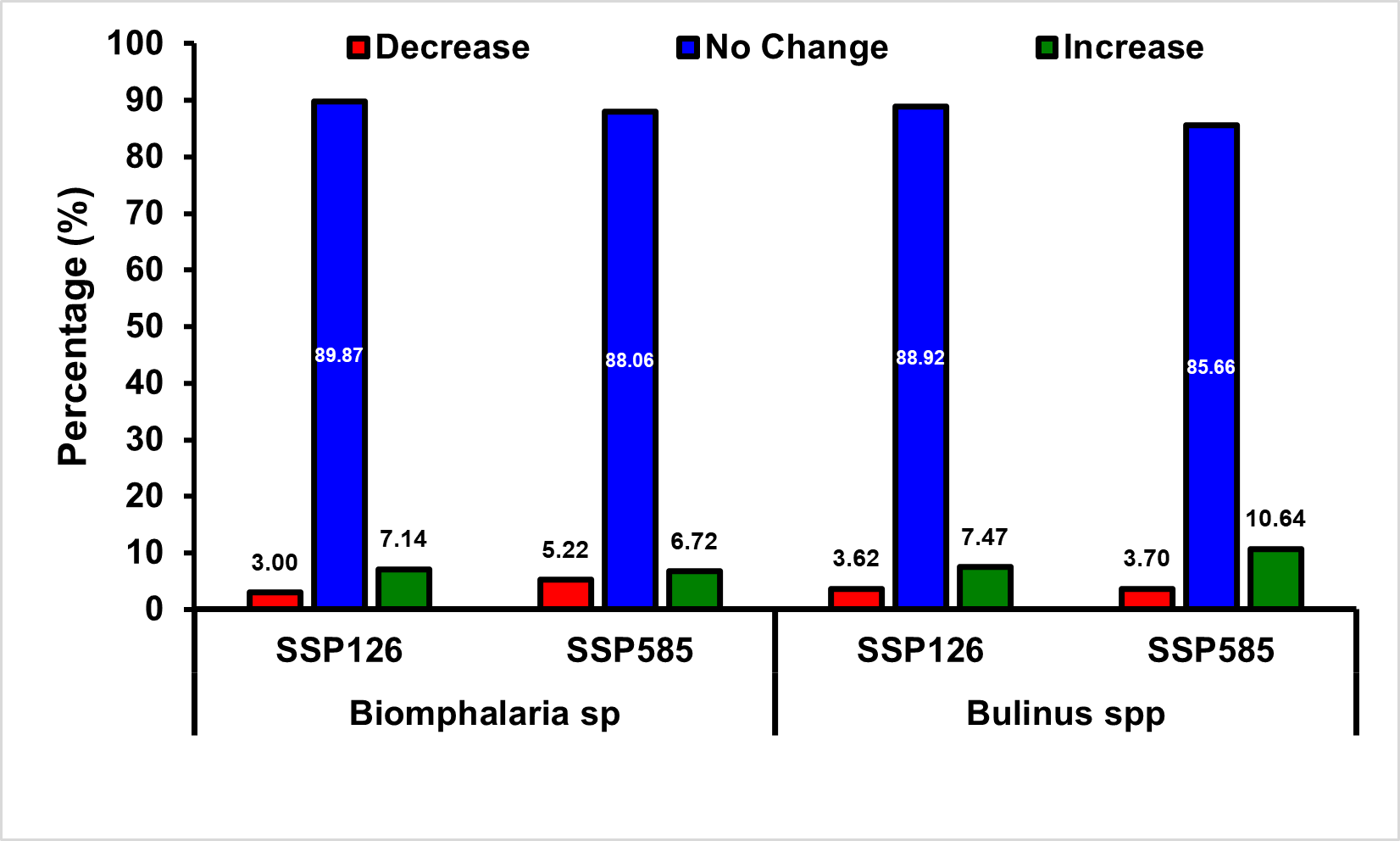


**Supplementary S1 Fig. 5**. Proportion of habitat suitability showing decrease (red), no change (blue), and increase (green) under current and future climate scenarios (SSP1-2.6: low emissions, SSP5-8.5: high emissions) for *Biomphalaria* and *Bulinus.*


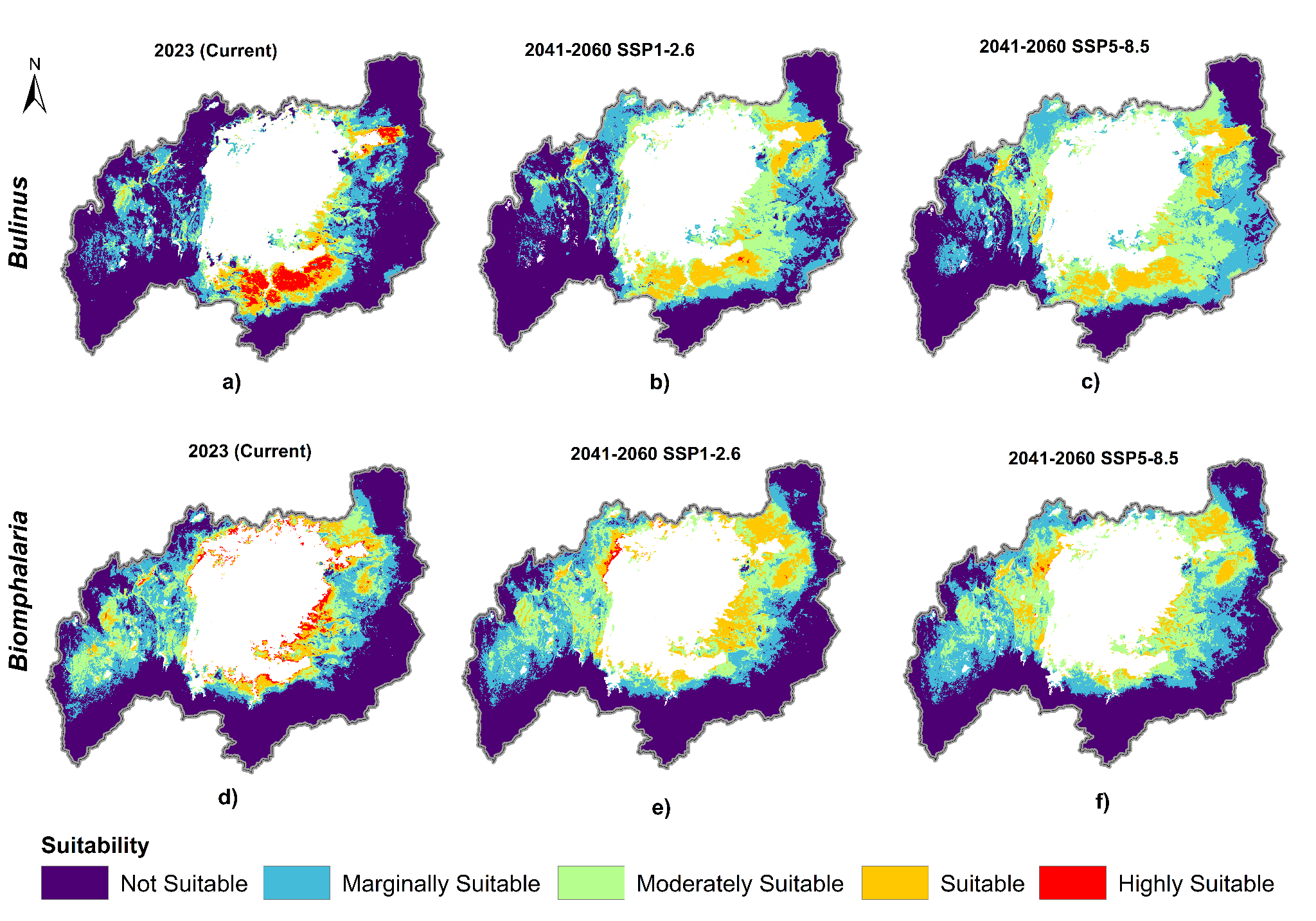


**Supplementary S1 Fig. 6.** Habitat suitability maps showing specific suitability categories (not suitable, marginally suitable, moderately suitable, suitable and highly suitable) for *Bulinus* (top panels) and *Biomphalaria* (bottom panels) genera in the Lake Victoria Basin under current conditions (first column: a, d) and projected future climate scenarios based on Shared Socioeconomic Pathways (SSPs) from 2041 to 2060. The middle column (b, e) represents a low-emission scenario (SSP1-2.6), while the last column (c, f) depicts a high-emission scenario (SSP5-8.5).

***
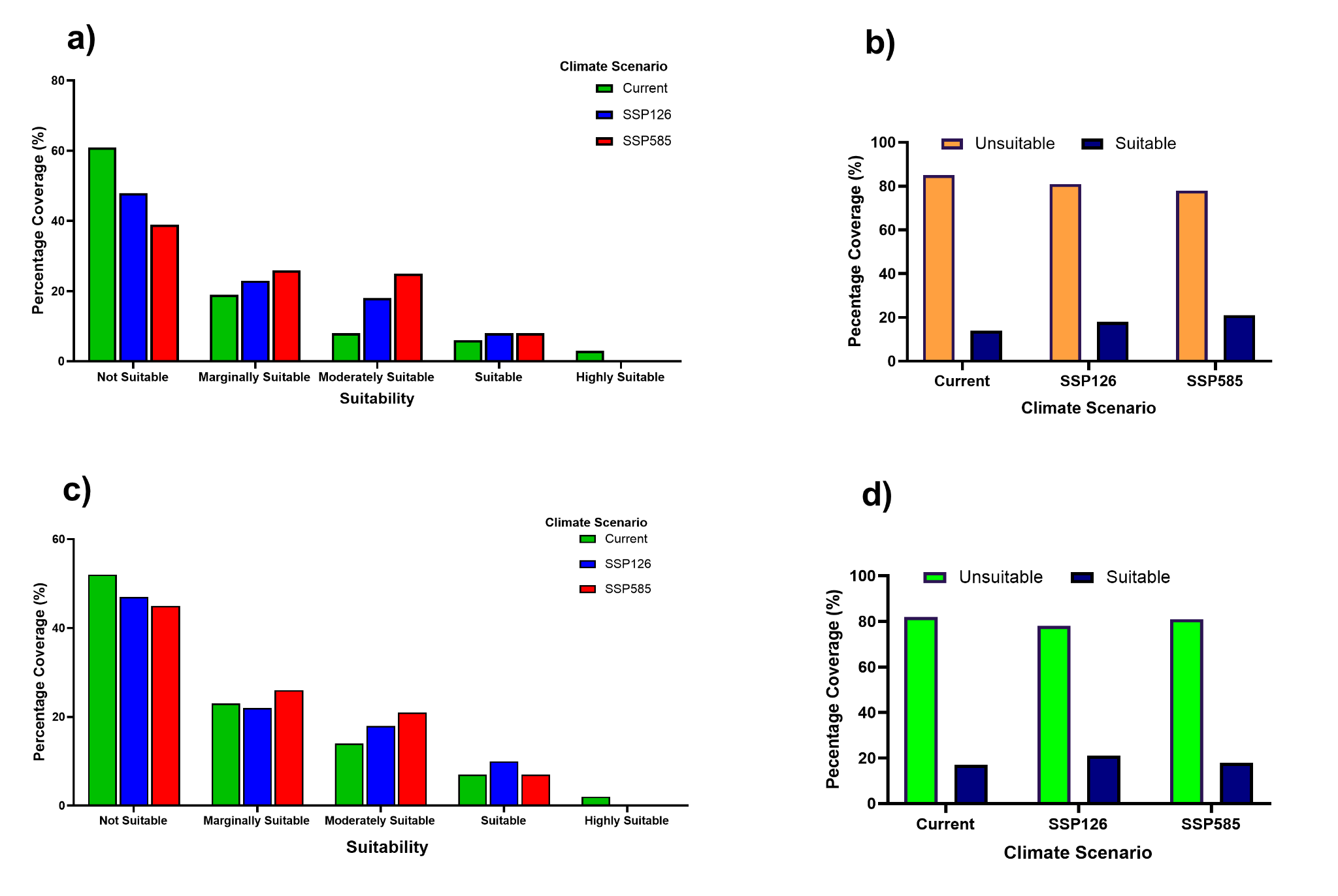
***

**Supplementary S1 Fig. 7:** Quantification of habitat suitability for *Bulinus* (a, b) and *Biomphalaria* (c, d) in the Lake Victoria basin (a, c): Bar graphs illustrating the proportion of habitat suitability (e.g., not suitable, marginally suitable, moderately suitable, suitable and highly suitable) under current conditions and future scenarios based on SSP1-2.6 (low emissions) and SSP5-8.5 (high emissions). (b, d): Bar graphs showing the distribution of classified habitat suitability (e.g., suitable and unsuitable) under current conditions for the respective species in the Lake Victoria basin.

**Supplementary S1 Table 3**: Habitat Suitability for *Bulinus* and *Biomphalaria* in the Lake Victoria Basin. The table presents proportional habitat suitability (suitable or unsuitable) under current conditions and future climate scenarios (SSP1-2.6: low emissions, SSP5-8.5: high emissions). It also details the distribution of classified habitat suitability categories (suitable and unsuitable).

| ***Bulinus* spp.** | | | | | | |
| --- | --- | --- | --- | --- | --- | --- |
| Suitability Class | **Climate Change Scenario** | | | | | |
|  | **Current** | | **SSP1-2.6** | | **SSP5-8.5** | |
|  | **Area (km^2^)** | **Coverage (%)** | **Area (km^2^)** | **Coverage (%)** | **Area (km^2^)** | **Coverage (%)** |
| Unsuitable | 190495 | 85.62 | 181933 | 81.77 | 175045 | 78.68 |
| Suitable | 31986 | 14.38 | 40551 | 18.23 | 47439 | 21.32 |
| ***Biomphalaria* spp.** | | | | | | |
| Unsuitable | 184528 | 82.94 | 175315 | 78.8 | 181194 | 81.44 |
| Suitable | 37953 | 17.06 | 47169 | 21.2 | 41290 | 18.56 |

**Supplementary S1 Table 4:** The table presents a comparative analysis of **current and future schistosomiasis exposure risks** under two climate scenarios: **SSP1-2.6 (low emissions, sustainable future)** and **SSP5-8.5 (high emissions, fossil-fuel-driven growth).** It also details the distribution of classified exposure risk categories (Very low, Low, Medium, High and Very high) under current and future conditions.

| **Risk** | **Intestinal Schistosomiasis Exposure Risk** | | | **Urogenital Schistosomiasis Exposure Risk** | | |
| --- | --- | --- | --- | --- | --- | --- |
|  | **Current** | **SSP1-2.6** | **SSP5-8.5** | **Current** | **SSP1-2.6** | **SSP5-8.5** |
|  | **Coverage (%)** | **Coverage (%)** | **Coverage (%)** | **Coverage (%)** | **Coverage (%)** | **Coverage (%)** |
| **Very low** | 52.7 | 48.81 | 45.09 | 62.01 | 49.99 | 41.06 |
| **Low** | 25.15 | 26.18 | 25.42 | 20.85 | 28.8 | 34.38 |
| **Medium** | 13.3 | 15.91 | 21.97 | 7.93 | 14.48 | 17.67 |
| **High** | 8.67 | 9.08 | 6.41 | 9.12 | 6.73 | 6.89 |
| **Very high** | 0.19 | 0.01 | 1.11 | 0.09 | 0 | 0 |

**References**

[1] L. Breiman, Random forests, Mach. Learn. 45 (2001) 5–32. https://doi.org/https://doi.org/10.1023/A:1010933404324.

[2] M. Kuhn, Caret: classification and regression training, Astrophys. Source Code Libr. (2015) ascl-1505.

[3] K.A. Nguyen, U. Seeboonruang, W. Chen, Projected Climate Change Effects on Global Vegetation Growth: A Machine Learning Approach, Environments 10 (2023) 204.
